# Differentiating DeepSeek AI-generated translations from NMT translations and human translations within Chinese white paper discourse: a computer-based method for analyzing writing style

**DOI:** 10.3389/fpsyg.2026.1931933

**Published:** 2026-10-26

**Authors:** Ruoyang Zhang, Xiaoting Ji, Jun Zou

**Affiliations:** 1 School of Foreign Languages, Tianjin Normal University, Tianjin, China; 2 School of Foreign Languages, Shaoxing University, Shaoxing, China

**Keywords:** cognitive load, computational stylistics, government white papers, human translation, LLM translation, multidimensional analysis, neural machine translation, psychology of language

## Abstract

This study investigates whether distinct “machine-translationese” and “LLM-translationese” exist in Chinese–English government translation by comparing DeepSeek V4Pro, Google neural machine translation, and pre-neural human translation. We built the corpus as 24 minimally overlapping 5,000-word windows per translation condition, yielding 72 matched observations. Using 21 normalized linguistic features selected exclusively within a group-aware training partition, five classifiers were trained and evaluated with a fully held-out, chunk-disjoint test set, and their stability was cross-checked with nested cross-validation and 50 repeated group-aware splits. Naive Bayes and Random Forest emerged as the most consistently above-chance classifiers, while the remaining classifiers were weaker or more sensitive to the specific partition. This indicates that the three translation agents are distinguishable from linguistic form alone. A five-factor multidimensional analysis identified discourse cohesion and causal-deictic linking as the strongest and most stable group contrast separating human translation from both machine systems, alongside four additional, comparatively weaker dimensions capturing nominal/lexical density, nominalization and wh-subordination, lexical diversity and dynamic aspect, and elaborative/predicative complexity. Corrected pairwise comparisons confirmed that human translation exceeds both machine systems on discourse cohesion, and that Google NMT and DeepSeek V4Pro are statistically indistinguishable from one another on most dimensions. A chunk-level bootstrap analysis confirmed that, in five-dimensional factor space, DeepSeek V4Pro and Google NMT sit reliably closer to each other than either does to human translation, supporting a shared machine-translationese distinct from institutional human translation. The study documents differences in stylistic profile and presents the proposed links to reader cognitive load and RLHF-driven hedging as hypotheses emerging from these structural findings. These results provide an empirically grounded, methodologically conservative basis for discussing LLM- and NMT-based translation in official contexts.

## 1 Introduction

Translationese, a core concept in translation studies established following the work of Baker (1993) on universal traits, refers to the unique linguistic style that emerges from the process of translating text. The widespread adoption of automated translation tools in recent years has expanded the concept of translationese into new areas. Prior research has established that the language produced by machine translation constitutes a distinct, recognizable variety. Scholars have referred to this phenomenon as “machine-translationese” (Läubli et al., 2018; Toral et al., 2018) or “MT-specific translationese” (Vanmassenhove et al., 2021). Neural machine translation (NMT) did not eliminate these markers; instead, it established its own distinctive stylistic profile, characterized by greater surface-level coherence, a reliance on predictable word combinations, and consistent grammatical structures that distinguish NMT output from both human-translated texts and original English prose (Bizzoni et al., 2020; Niu and Jiang, 2024).

The rise of large language models (LLMs) as translation agents marks a new and theoretically important shift in this trajectory. Architecturally, models such as ChatGPT, DeepSeek, Gemini, and their successors differ from traditional neural machine translation systems at a foundational level. Although encoder–decoder models, like those powering Google Translate, are trained on matched bilingual texts for precise sentence-level translation, decoder-only large language models undergo a different process; they are first trained on enormous and varied, mainly web-based data, before being refined through reinforcement learning based on human feedback (RLHF) to function as interactive conversational tools.

This architectural distinction is not only technical—it corresponds to two different accounts of how the output text is generated, and those two accounts have different psychological grounding. In cognitive models of writing (Flower and Hayes, 1981; Kellogg, 1996), professional human translation involves ongoing cognitive monitoring of the evolving target text, working-memory allocation across planning, translating, and reviewing sub-processes, and deliberate risk management around register, tone, and institutional accountability. A decoder-only LLM fine-tuned via RLHF approximates a third, distinct process again: rather than modeling either human translator cognition or reference-matching, it is optimized toward outputs that human raters judge helpful, fluent, and engaging in a general conversational sense—an objective with no built-in sensitivity to register-specific communicative norms. Whether LLM translations represent a distinct stylistic form—a recognizable “LLM-translationese”—remains a matter for empirical investigation.

Researchers have begun to address this question, though progress remains inconsistent. Research comparing large language model and neural machine translation outputs has predominantly relied on automated evaluation metrics (e.g., BLEU, COMET, chrF) or limited human assessments (Freitag et al., 2022; Hendy et al., 2023). When stylometric analysis has been used, studies have usually concentrated on a limited set of surface-level measures—such as type-token ratio, average sentence length, and readability scores—rather than the broader multi-feature profiling that corpus stylistics has demonstrated to be methodologically necessary (Biber, 1989). Most earlier work in this area has focused on literary, journalistic, or general translation. In contrast, the field of Chinese government communication—an area characterized by distinct conventions, major political implications, and well-documented institutional norms (Sheng and Kong, 2023; Cao et al., 2025; Jiang et al., 2026)—has received considerably less systematic attention.

This is exactly the area where stylistic differences are most consequential, and not only in a diplomatic sense. Register violations in political white papers—inappropriate hedging where firm commitment is expected, or informal pragmatic markers where institutional distance is expected—do not simply “read oddly.” Rather, they are theoretically expected to increase the processing load placed on international readers, who must do additional inferential work to reconcile an unexpected register with the institutional source of the text; because the present study includes no reader experiment, we treat this cognitive-load implication as a hypothesis rather than an established result. Furthermore, they can shift how confidently readers attribute a stated position, commitment, or belief to the issuing government.

In this context, the study uses a specially compiled, sentence-aligned corpus of Chinese-to-English translations of white papers from the State Council Information Office to examine stylistic variations among translations generated by DeepSeek V4Pro, Google Neural Machine Translation, and human translators in an institutional setting.

## 2 Literature review

### 2.1 Comparative analysis of translation versions: human, NMT, and LLM translations

#### 2.1.1 The distinctive stylistic features of neural machine translation

The stylistic features of NMT output have received significant academic interest since the introduction of attention-based NMT (Bahdanau et al., 2014), especially after the broad adoption of Transformer-based models (Vaswani et al., 2017). Initial research using text corpora showed that while neural machine translation produces more fluent output than statistical machine translation, it consistently displays linguistic patterns that are absent from both original English writing and human translations. Jiang and Niu (2022) analyzed a large English-Chinese parallel corpus and showed that NMT depends more on superficial cohesive markers than human translations do, leading to text that is formally coherent but lacks pragmatic depth. Loock (2020) likewise found NMT texts lexically simpler than human translations, showing lower type-token ratios, reduced lexical diversity, and a tendency to use high-frequency vocabulary.

Sheng and Kong (2023) looked at machine translations of Chinese political documents and found that these systems consistently fail to capture the communicative authority that institutional language carries—including the ability to indicate what an institution knows or believes, assign accountability, and adjust how strongly claims are expressed to fit diplomatic communication contexts. Bizzoni et al. (2020) demonstrated that texts produced by Neural Machine Translation across several European language pairs are distinguishable from human translations when assessed using readability metrics and dependency-based complexity measures, indicating that the architectural constraints of encoder–decoder NMT systems result in a consistent stylometric signature across various domains and language pairs.

#### 2.1.2 Translation using large language models: quality, competence, and evolving characterization

Hendy et al. (2023) compared several early GPT models with WMT-Best systems and Microsoft Translator across high-resource and low-resource language pairs. Results showed that for high-resource languages, GPT models achieved very competitive translation quality, while their performance was more limited for low-resource languages. DeepSeek models have more recently drawn attention for translation capabilities comparable to Google Translate and earlier GPT versions. Al Rousan et al. (2025) noted consistent shortcomings in LLMs’ ability to manage cultural subtleties and pragmatic norms in Arabic-to-English translation, including a tendency to translate idioms too literally and a failure to appropriately adjust linguistic register—which are limitations of particular significance in institutionally regulated domains.

Chen and Hong (2025) investigated LLM translation of Chinese legal texts and found that LLMs had difficulty consistently employing the complex syntactic structures required by legal language; these structures include conditional clauses, stacked relative clauses, and passive constructions. Yao and Fan (2025) developed and validated a Cognitive Load Scale for AI-assisted L2 writing, and they established that interacting with AI-generated text imposes measurable, structured cognitive strain on the human reader or reviser.

### 2.2 Multi-feature methodological frameworks

#### 2.2.1 Multidimensional approaches to translation stylistics

Biber's (1989) multidimensional analysis (MDA) framework, which uses factor analysis to detect patterns of co-occurring linguistic features and uncovers the functional dimensions driving variation in language use, provides the theoretical foundation for this study.

Hu et al. (2019) set a standard for MDA-based research on translated English. They demonstrated that a corpus of Chinese–English translations can be reliably distinguished from original English texts in terms of informational detail, interactive engagement, and narrative focus. Li and Hu (2024) pointed out the tension between normalization, where translated texts align with target-language norms, and “shining through,” where source-language structural features persist in translation; they argued that identifying these opposing influences requires analyzing multiple language features at the same time. This “shining through” tension is directly relevant to interpreting why machine output in the present corpus sometimes shows higher nominal/lexical density than human translation, contrary to a naive “machines simplify” expectation.

#### 2.2.2 Extracting key features and the study of translation

The range and theoretical basis of linguistic features employed in MDA-based translation research have undergone significant development. The MFTE tagger (Le Foll and Shakir, 2025), an expanded version of the MAT tagger (Nini, 2019) that adds semantic and functional tagging categories from Biber (1992) and Biber (2006), enables extraction of more than 130 features across lexical, syntactic, semantic, and discourse levels—including modal verb subtypes, pronoun categories, subordination types, discourse connectives, and semantic categories of verbs, adjectives, and adverbs.

#### 2.2.3 Research gap and research questions

Although research on NMT stylometry is increasing and studies on LLM translation quality are emerging, no prior study has used a comprehensive multi-feature MDA framework, cross-validated supervised classification, and a psychologically grounded interpretive lens together to compare translations produced by LLMs, NMT systems, and human translators within Chinese government written discourse. Because official government discourse constrains modality, stance, and intent through institutional norms, diplomatic protocols, and ideological frameworks—this domain is an ideal context for identifying translation-agent-specific stylistic patterns and for asking what those patterns cost readers in processing terms.

Building on the literature reviewed and the identified gaps, the present study addresses three research questions:

RQ1 (Distinguishability): Are machine-learning classifiers capable of reliably distinguishing among the three translation variants using exclusively linguistic features, and if so, which features and which classifiers provide the most trustworthy evidence of this distinction?

RQ2 (Functional Profile): What specific linguistic features does multidimensional analysis identify across the three translated versions, how robust these functional dimensions are to the way discriminating features are selected, and which of these dimensions plausibly carry the greatest processing and stance-attribution consequences for readers?

RQ3 (LLM-Specificity): Within the stylometric framework of multiple dimensions, does the translation produced by DeepSeek V4Pro align more closely with human translation or with Google Neural Machine Translation, and is the apparent gap between machine-translation pairs and human translation statistically reliable?

## 3 Methodology

We used a multi-feature approach combining confirmatory (machine-learning classification) and exploratory (multidimensional factor analysis) methods to analyze normalized linguistic features taken from a rolling stylometry corpus of matched, minimally overlapping text windows. The complete analysis pipeline is documented in Appendix D.

### 3.1 Developing the corpus and dividing the texts

#### 3.1.1 Sub-corpora

The corpus contains three parallel, sentence-aligned sub-corpora; each sub-corpus holds a complete Chinese-to-English translation of the same series of white papers issued by the State Council Information Office (Appendix A): Throughout this article, NMT denotes the Google NMT sub-corpus rather than neural machine translation as a technology.

- Human Translation (HT): This sub-corpus contains English translations created by institutional translators employed or contracted by the official publishing system of the Chinese government. All human translations in this corpus were completed before 2013, predating the broad accessibility of commercial neural machine translation and large language model tools. The chronological cut-off ensures that the HT sub-corpus reflects genuine pre-neural institutional practices, free from the standardizing effects of NMT post-editing or prompt-driven revisions. Sub-corpus statistics: 7,140 types, 109,335 tokens.
- Google NMT (NMT): English translations produced by Google Translate during 2026, using a transformer-based neural machine translation system trained on extensive multilingual parallel datasets. Sub-corpus statistics: 6,055 types, 109,130 tokens.
- DeepSeek V4Pro (V4Pro): English translations were produced in 2026 using the DeepSeek V4Pro API, a decoder-only large language model blending dense and mixture-of-experts layers, trained on extensive multilingual web data and refined through RLHF for dialogue-oriented tasks. Sub-corpus statistics: 6,298 types, 108,274 tokens.

The source corpus comprises Chinese white papers, containing 8,617 unique word types and 72,745 total tokens. The three translation sub-corpora contain a roughly equal number of tokens (108,000–109,500 each), ensuring comparability at the overall level. Because all three sub-corpora are translations of the same underlying Chinese source sequence, the corpus enables a paired, source-controlled comparison across translation agents (Section 3.1.3).

#### 3.1.2 Procedure for generating machine translations and source segmentation

The two sets of machine translation data were produced under controlled experimental settings to ensure comparability and replicability, and both were generated at the level of the complete white-paper document rather than from isolated sentences or paragraphs, so that document-level context was available to both machine systems during generation.

Google Neural Machine Translation was accessed via its web-based interface with default settings; this interface exposes no configurable system-prompt layer. Each white paper was submitted as a complete document; Google’s output maintains a direct line-by-line correspondence with the Chinese source, meaning each source line maps onto a single, identifiable output line.

For DeepSeek V4Pro, translations were produced through the official API, document by document. Within each document, the Chinese source was first split into sentences and each sentence was numbered; the prompt instructed the model to translate every numbered sentence and to return the translations strictly sentence-by-sentence under the same numbering, without merging, splitting, omitting, adding, or reordering numbered items, and without any content outside the numbered list. A representative call can be found in Appendix E.

All calls used a single-turn session with no dialogue history retained across documents, and we enabled extended reasoning (“thinking”) with reasoning_effort = “high.” Apart from temperature = 0, decoding parameters were not explicitly overridden and therefore followed the API’s default values. Beyond the client library’s generic default system message (“You are a helpful assistant”), we supplied no task-specific instruction, style guide, or safety-guardrail text. We used all machine-generated texts in their original form, without any subsequent editing.

Because Google’s output is aligned line-by-line with the source and DeepSeek’s output is constrained to a checkable, one-to-one numbered correspondence with source sentences, both machine sub-corpora retain an explicit sentence-level mapping to the same Chinese source used for the human translation, while still being generated with full document-level context available to the model.

#### 3.1.3 Rolling stylometry and cross-condition alignment

Using the rolling stylometry method developed by Eder (2016) to separate translational style from influences related to text length and subject matter, we treated each sub-corpus as a single, uninterrupted flow of text; this approach avoids analyzing individual documents, as that would mix stylistic signal with topic-related variation across the diverse white paper subjects.

Because the three sub-corpora are sentence-aligned translations of the same source, windows are defined on the HT sub-corpus. This sub-corpus was chosen as the reference series because human translation preserves the most conventional sentence-to-paragraph correspondence with the Chinese source. A 5,000-word window with a 4,500-word step is used, leaving an overlap of at most 500 words between neighboring windows (455–498 words in the present corpus). The Chinese source span underlying each HT window boundary is then used to mark out the matching window in the NMT and V4Pro sub-corpora, so that windows sharing a chunk identifier (ChunkID) correspond to the same portion of source text across all three conditions. This yielded 24 chunk positions with a complete matched set across HT, NMT, and V4Pro (72 windows in total: 24 per condition). Each window contains roughly the same number of tokens, reducing length-normalization issues, and the minimal-overlap structure sharply reduces the share of text any two neighboring windows have in common.

#### 3.1.4 Diachronic stability check

Because the HT sub-corpus predates the NMT and V4Pro sub-corpora by more than a decade, a possible confound exists. Observed HT–machine differences may partly reflect diachronic language change in institutional English rather than genuine agent-specific stylistic differences. To assess this directly, we compiled an additional post-2014 institutional human translation corpus (59 chunks) and compared the two periods on the 136 features shared with the main analysis, using Mann–Whitney U tests with Bonferroni correction.

None of the 136 shared features differed significantly between the two periods after Bonferroni correction (Table 1). The largest differences were declines in discourse particles (RP, −27.6%), stance adverbs (RSTNCall, −62.4%), non-factive stance adverbs (RNONFACT, −64.2%), and topical adjectives (JJTOPIC, −29.7%), together with an increase in quantifier frequency (QUAN, +21.9%); none of these, nor any other feature, reached the corrected significance threshold. In particular, none of the features that structure our factor solution (Section 4.2) shifted significantly.

**Table 1:** Diachronic comparison: pre-2013 HT vs. post-2014 HT.

Feature	Mean (pre-2013)	Mean (post-2014)	Change (%)	*p*_adj_
RP (discourse particles)	0.360	0.261	−27.6%	0.064
RSTNCall (stance adverbs, all)	0.052	0.020	−62.4%	0.108
RNONFACT (non-factive stance adverbs)	0.042	0.015	−64.2%	0.136
JJTOPIC (topical adjectives)	1.020	0.717	−29.7%	0.501
NNABSPROC (abstract/process nouns)	7.237	6.123	−15.4%	0.532
QUAN (quantifiers)	0.714	0.870	+21.9%	0.583

We see this pattern as evidence for considerable stylistic stability over time in institutional diplomatic translation: the largest feature-level differences involve stance-adverbial and topic-linked categories, and none survives correction for multiple comparisons. We therefore treat the pre-2013 HT sub-corpus as a defensible, though not unconfounded, human-translation baseline, and we return to this issue in the Limitations (Section 6.1).

### 3.2 Extraction of linguistic features

Features were identified using the MFTE tagger (Le Foll and Shakir, 2025), which integrates part-of-speech tagging with semantic and functional annotation categories based on Biber (1992) and Biber (2006). For each text window, the tagger generates 142 features—141 linguistic features plus the raw word count of each window—spanning lexical (e.g., average word length, type-token ratio, lexical density), grammatical (e.g., passive constructions, progressive aspect, split infinitives), pronominal, modal (possibility, necessity, conditional, predictive), syntactic complementation (that-clauses, wh-clauses, to-infinitival complements), etc. All linguistic features were normalized per 1,000 words to control for window-size variation.

### 3.3 Selection of features

Since the 72 windows are drawn from three matched sub-corpora describing the same underlying white-paper series, the appropriate univariate test for identifying candidate discriminating features is the paired (Friedman) test. Because the same feature-selection procedure feeds both an exploratory dimensional analysis (Section 3.5) and a supervised classification task whose evaluation must remain uncontaminated by the held-out test partition (Section 3.4), we ran two separate feature-selection pipelines.

Exploratory (MDA) feature set: Each of the 142 tagger outputs was tested with a paired Friedman test across the three translation types using the full 72-window sample, with Bonferroni correction for multiple comparisons; 30 features reached significance (adjusted *p* < 0.05). Absolute Pearson correlation coefficients were then calculated between every pair of the 30 significant features, and pairs with |*r*| > 0.8 were treated as redundant, with the feature having the weaker Kendall’s W dropped from each pair. This step removed six features—IN, JJATother, PP1all, PP3t, RBother, and WHSCother—resulting in a final set of 24 features used exclusively for the exploratory multidimensional analysis detailed in Section 4.2. Because this feature set is selected using the same three-way group contrast that is later tested for significance at the dimension level, we treat the resulting MDA as exploratory and report an independent, non-circular validation check in Section 4.2.8.

Classification feature set: To avoid the held-out test partition influencing feature selection, a second, independent Friedman-test-plus-correlation-pruning procedure was applied using only the 57 windows in the training partition (Section 3.4): 27 features reached Bonferroni-corrected significance within the training data alone, and the same |*r*| > 0.8 correlation-pruning step removed the same six redundant features (IN, JJATother, PP1all, PP3t, RBother, WHSCother), leaving 21 features. These 21 training-only-selected features, and no others, were used to train and evaluate every classifier reported in Section 4.1; the test partition played no role in choosing them. Both feature sets are listed in Appendix B.

### 3.4 Machine learning-based classification

Group-aware train/test split: Because neighboring windows still share a modest 500-word overlap, a conventional stratified split risks placing near-duplicate windows on both sides of the partition. We therefore used a group-aware split (GroupShuffleSplit, test_size = 0.2, random_state = 42) that keeps every window sharing a ChunkID entirely within either the training or the test partition. From this process, 57 training windows and 15 test windows were produced; these corresponded to 19 and 5 chunks respectively, with no ChunkIDs shared between the two partitions.

Classifiers: The resulting matrix of 57 training windows by 21 features (Section 3.3) served to train and evaluate five classifiers: a Linear Support Vector Machine (SVM), a Random Forest (RF), a Multilayer Perceptron (MLP), AdaBoost, and Gaussian Naive Bayes.

- Linear SVM: C = 0.025, kernel = linear, decision_function_shape = ovr, gamma = scale, tol = 0.001, random_state = 42.
- Random Forest: n_estimators = 10, max_depth = 5, max_features = 1, criterion = gini, bootstrap = True, random_state = 42.
- MLP (Neural Net): hidden_layer_sizes = (100,), activation = relu, solver = adam, alpha = 1.0, learning_rate = constant, learning_rate_init = 0.001, max_iter = 1,000, early_stopping = False, random_state = 42.
- AdaBoost: n_estimators = 50, learning_rate = 1.0, algorithm = SAMME. R, random_state = 42.
- Naive Bayes: var_smoothing = 1 × 10^−9^, priors = None.

Model assessment used 5-fold GroupKFold cross-validation within the 57-window training partition, again grouped by ChunkID, so no fold ever places a training and validation window from the same source position on opposite sides of a fold boundary. Each fitted model was evaluated exactly once on the untouched 15-window test set, reporting both CV accuracy (mean ± SD across folds) and test-set accuracy, precision, recall, and F1 (Section 4.1, Table 2).

**Table 2:** Classification performance of five classifiers.

Classifier	CV accuracy (Mean ± SD)	Test accuracy	Test precision (macro)	Test recall (macro)	Test F1 (macro)	Test F1 (micro)
Linear SVM	0.506 ± 0.087	0.600	0.571	0.600	0.555	0.600
Random Forest	0.728 ± 0.102	0.867	0.867	0.867	0.867	0.867
MLP (Neural Net)	0.400 ± 0.062	0.400	0.452	0.400	0.287	0.400
AdaBoost	0.478 ± 0.159	0.400	0.485	0.400	0.319	0.400
Naive Bayes	0.833 ± 0.105	0.800	0.822	0.800	0.805	0.800

Robustness checks: Three additional analyses probe how sensitive these results are to the specific train/test partition and to residual overlap between neighboring chunks. First, a nested cross-validation was run in which the 21-feature classification set was re-selected independently inside every outer fold, so that no fold’s validation rows could influence which features were available to classify it. Second, the group-aware 80/20 split (with feature re-selection on each training partition) was repeated 50 times with different random seeds, to characterize the sampling variability that a single split necessarily obscures. This repeated-split evaluation was re-run with a one-chunk buffer that additionally excludes, from the training set only, any chunk adjacent to a chunk placed in that repetition’s test set. This serves as a conservative guard against the residual 500-word overlap between neighboring chunks. The reports for all three checks are presented in Section 4.1 (Table 3).

**Table 3:** Classification robustness checks.

Classifier	Nested CV accuracy (Mean ± SD)	Repeated split, no buffer (Mean ± SD, 50 shuffles)	Repeated split, 1-chunk buffer (Mean ± SD, 50 shuffles)
Linear SVM	0.489 ± 0.057	0.572 ± 0.094	0.484 ± 0.092
Random Forest	0.606 ± 0.106	0.753 ± 0.088	0.623 ± 0.128
MLP (Neural Net)	0.683 ± 0.213	0.596 ± 0.201	0.579 ± 0.176
AdaBoost	0.456 ± 0.154	0.491 ± 0.169	0.561 ± 0.127
Naive Bayes	0.794 ± 0.078	0.811 ± 0.095	0.667 ± 0.112

### 3.5 Multidimensional analysis

At the exploratory stage (RQ2), we used a six-step MDA approach based on Biber (1989), deriving dimensions empirically instead of taking Biber’s pre-established dimensions, because domain-specific corpora like this one can have factor structures that differ from cross-genre reference corpora. The six stages were: (1) selecting features as described in Section 3.3; (2) identifying the number of factors to retain by examining the scree plot and an automated Kneedle elbow-detection algorithm; (3) extracting factors via principal-axis factoring with varimax rotation; (4) deriving factor loadings; (5) interpreting each factor functionally as a dimension of variation; (6) calculating dimension scores per window and comparing them across translation types.

The five-factor solution that was kept accounts for 59.2% of total variance (Table 4). Features with absolute loadings ≥ 0.30 were used to interpret each dimension.

**Table 4:** Proportion of variability accounted for by five factors, and dimension-level significance.

Factor	SS loadings	Variance (%)	Cumulative (%)	Friedman *χ*^2^	Kendall’s *W*	Sig.
D1	3.951	16.5	16.5	15.75	0.328	***
D2	2.904	12.1	28.6	7.75	0.161	*
D3	2.845	11.9	40.4	39.08	0.814	***
D4	2.345	9.8	50.2	6.25	0.130	*
D5	2.154	9.0	59.2	7.58	0.158	*

***p < 0.001, *p < 0.05 (paired Friedman test).

The same repeated-measures structure applies to the 72 dimension-score observations, so dimension-level group comparisons use the paired Friedman test, with Kendall’s W reported as the paired-design effect size, followed by Holm-corrected Wilcoxon signed-rank post-hoc tests for each pairwise contrast (Section 4.2.9). Appendix C gives further technical detail. A concern is that features selected because they differ across the three translation conditions cannot straightforwardly be re-tested for differing across those same conditions without some circularity; Section 4.2.8 therefore reports a separate validation analysis in which the factor structure is re-derived from an independently, non-circularly selected feature subset and compared to the main solution via Procrustes matching.

### 3.6 Distance calculation and t-SNE visualization

To address RQ3, Euclidean distances were calculated between all pairs of HT, NMT, and V4Pro windows, using the five dimension scores from the factor analysis as input vectors. For each pair, the study reports mean distance and standard deviation, together with a chunk-level bootstrap (5,000 resamples, resampling whole ChunkIDs rather than individual windows to respect the paired design) providing 95% confidence intervals both for each pairwise mean distance and for the differences between distances. To visualize distance structure, t-SNE (perplexity = 30, random_state = 42) projected the five-dimensional score matrix into two dimensions, with two-standard-deviation confidence ellipses plotted per translation type.

## 4 Results

### 4.1 Classification using machine learning (RQ1)

Cross-validated and held-out test performance for all five classifiers on the leakage-controlled, group-aware split is reported in Figure 1 and Table 2. Naive Bayes was the strongest and most consistent classifier (CV accuracy = 0.833 ± 0.105; test accuracy = 0.800, macro-F1 = 0.805), followed by Random Forest (CV = 0.728 ± 0.102; test accuracy = 0.867, macro-F1 = 0.867). Linear SVM performed modestly above the 33.3% chance baseline in both cross-validation (0.506 ± 0.087) and on the test set (0.600). MLP and AdaBoost, by contrast, performed near or at chance on this particular held-out split (test accuracy = 0.400 for both); nonetheless, as the robustness checks below show, this single-split result understates their performance under other evaluation schemes.

**Figure 1:**
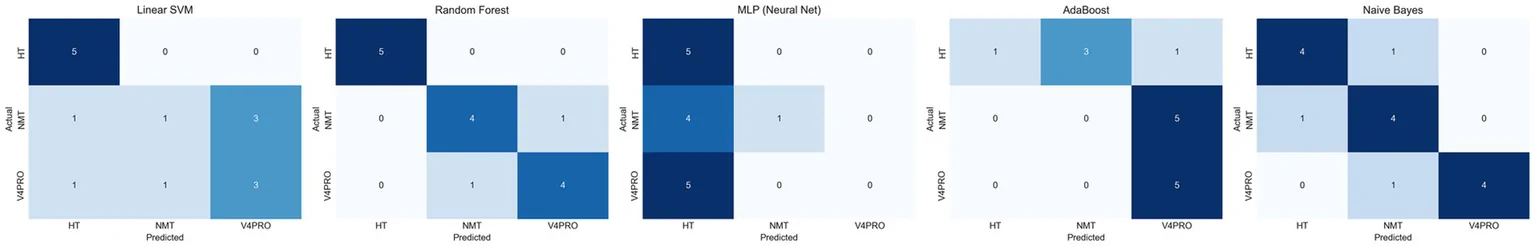
Confusion matrices of five classifiers for distinguishing among HT, NMT, and V4Pro translations.

Table 3 reports three robustness checks: a nested cross-validation that re-selects features inside every fold, and 50 repeated group-aware splits evaluated with and without a one-chunk training buffer around each test chunk. Across all three checks, Naive Bayes and Random Forest remain the most dependably above-chance classifiers (Naive Bayes: 0.667–0.811; Random Forest: 0.606–0.753), while Linear SVM stabilizes in the 0.48–0.57 range. MLP’s apparently weak single-split test performance does not generalize: its nested-CV accuracy (0.683) and repeated-split means (0.596 without buffer, 0.579 with buffer) are both well above the single-split figure, though its variability across repetitions is also the highest of any classifier (SD up to 0.201), which fits with a decision boundary that is sensitive to which chunks happen to fall in a given training set. AdaBoost is the weakest and most consistently unstable classifier throughout (0.456–0.561). Introducing the one-chunk buffer—which removes, from training only, any chunk immediately adjacent to a test chunk—modestly reduces accuracy for most classifiers (most visibly Random Forest and Naive Bayes), fitting with a small residual contribution from the remaining 500-word inter-chunk overlap.

These results collectively support a qualified answer to RQ1: linguistic form carries genuine, above-chance information about translation agent—most reliably so under Naive Bayes and Random Forest, and to a lesser and more variable extent under the other three classifiers.

### 4.2 Multidimensional analysis (RQ2)

#### 4.2.1 Suitability of factors and their extraction

Adequacy tests indicated that the correlation matrix was suitable for factor analysis (Table 5) (Kaiser, 1974). The Kneedle elbow-detection algorithm, applied to the exploratory 24-feature set (Section 3.3), found five factors to be the optimal number to retain (Figure 2).

**Table 5:** Factor adequacy statistics.

Statistic	Value
KMO measure of sampling adequacy	0.602
Bartlett’s test of sphericity (*χ*^2^)	Significant
Bartlett’s *p*-value	<0.001

**Figure 2:**
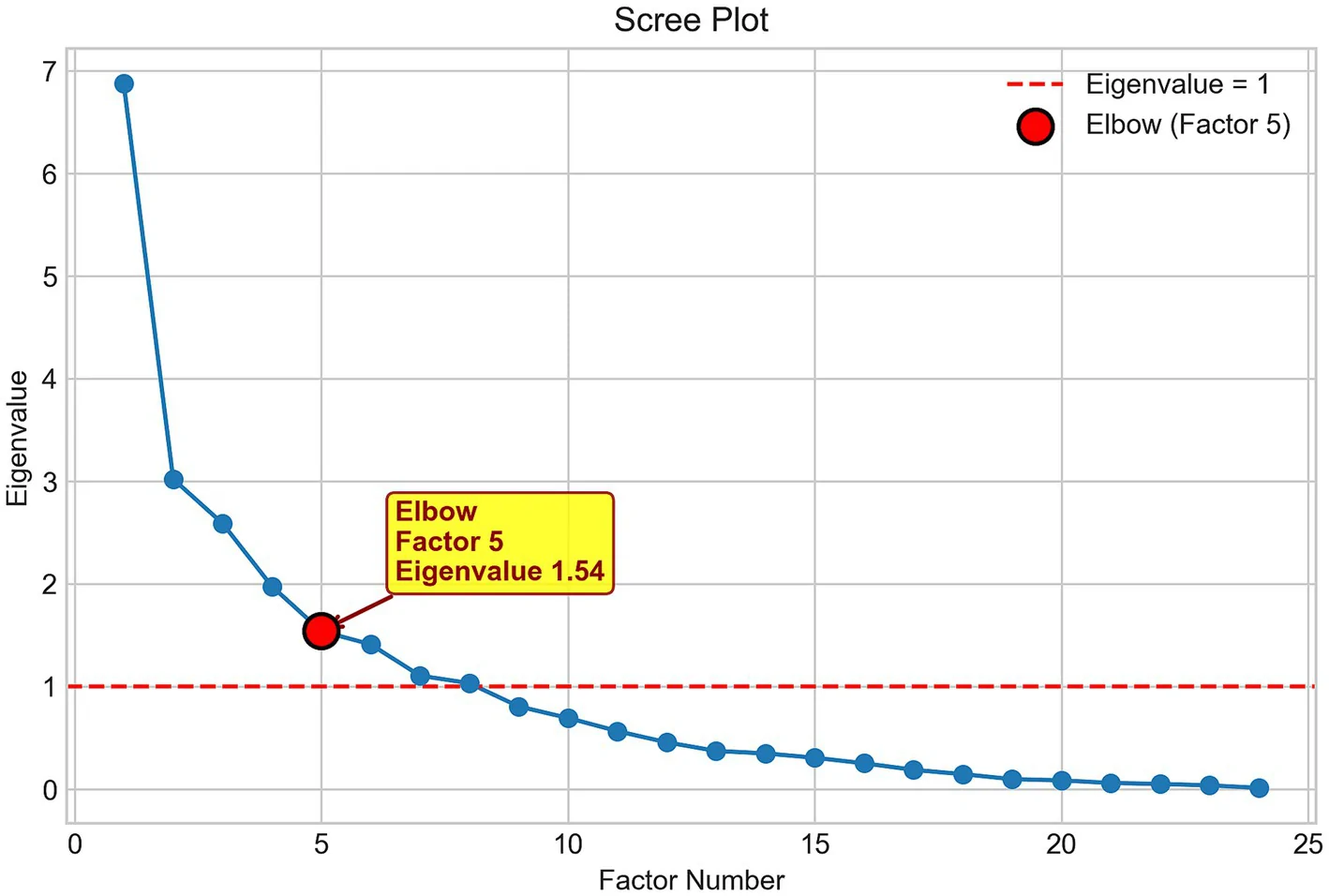
Scree plot and Kneedle elbow detection for the retained factor solution.

All five dimensions (Table 4) are significant under the paired Friedman test, but not uniformly so. D1 and D3 remain significant at *p* < 0.001, while D2, D4, and D5 are significant only at the conventional *p* < 0.05 level and carry small-to-moderate effect sizes. D3 (0.814) is by a wide margin the strongest and most robust dimension in the solution when ranked by effect size (Kendall’s W). It is followed at a considerable distance by D1 (0.328), D2 (0.161), D5 (0.158), and D4 (0.130).

#### 4.2.2 Dimension 1: nominal and lexical elaboration

Dimension 1 is defined by nominal compounds (NCOMP; *λ* = 0.859), a residual verbal/stance category (VIMP; *λ* = 0.752), average word length (AWL; *λ* = 0.740), lexical density (LDE; *λ* = 0.727), common nouns (NN; *λ* = 0.558), general adverbs (RB; *λ* = 0.429), coordinating conjunctions (CC; *λ* = 0.360), a residual predicative-adjective category (JJPRother; *λ* = 0.312), and first-person plural pronouns (PP1P; *λ* = 0.303).

This dimension measures overall nominal and lexical density: it indexes longer, more compound-heavy, more lexically dense noun phrases that are coordinated rather than subordinated. Corrected pairwise comparisons (Section 4.2.9) show that human translation scores significantly lower on this dimension than both machine systems, particularly V4Pro.

#### 4.2.3 Dimension 2: nominalization, third-person reference, and Wh-subordination

Dimension 2 is defined by nominalizations (NOMZ; *λ* = 0.767), all third-person pronouns (PP3all; *λ* = 0.709), wh-relative subordination (WHSC; *λ* = 0.634), and a residual preposition category (INother; *λ* = 0.473).

This dimension captures a cluster of features associated with abstract, impersonal reference and relative-clause elaboration. It is the third-strongest dimension (*χ*^2^ = 7.75, *p* = 0.021, Kendall’s *W* = 0.161); pairwise comparisons show human translation scoring significantly higher than NMT, though the HT–V4Pro contrast does not survive Holm correction.

#### 4.2.4 Dimension 3: discourse cohesion and causal-deictic linking

Dimension 3 is shaped by discourse particles (RP; *λ* = 0.685), it-cleft constructions (PIT; *λ* = 0.654), causal conjunctions (CUZ; *λ* = 0.614), demonstratives (DEMO; *λ* = 0.522), and activity verbs (ACT; *λ* = 0.398).

This dimension is by far the strongest in the solution (*χ*^2^ = 39.08, *p* < 0.001, Kendall’s *W* = 0.814)—causal conjunctions, resultative/discourse particles, and it-cleft emphasis together index explicit, argumentatively structured cohesion. HT consistently employs a denser network of explicit cohesive and deictic linking devices than either machine system. This ordering is preserved and further differentiated (NMT > V4Pro) under corrected pairwise testing (Section 4.2.9). Under the independent, non-circular validation check reported in Section 4.2.8, the discourse-cohesion contrast remains robust—it stays significant after Procrustes matching—whereas the factor loading structure of Dimension 3 does not: this dimension returns the lowest one-to-one congruence coefficient of the five (0.382), indicating that its loading pattern is comparatively sensitive to which features enter the analysis. Consequently, our greatest confidence is in the observed group contrast in discourse cohesion rather than in the stability of the Dimension 3 factor structure.

#### 4.2.5 Dimension 4: lexical diversity and dynamic aspect

Dimension 4 is driven by type-token ratio (TTR; *λ* = 0.738), activity verbs (ACT; *λ* = 0.568), progressive aspect (PROG; *λ* = 0.498), a residual preposition category (INother; *λ* = 0.358), and nominal compounds (NCOMP; *λ* = 0.306).

Texts scoring highly on this dimension combine greater lexical variety with a preference for presenting actions as ongoing rather than completed. As the weakest dimension in the solution (*χ*^2^ = 6.25, *p* = 0.044, Kendall’s *W* = 0.130), it shows HT scoring significantly higher than both machine systems, while NMT and V4Pro do not differ significantly from one another.

#### 4.2.6 Dimension 5: elaborative connection and predicative/split complexity

Dimension 5 is defined by a residual predicative-adjective category (JJPRother; *λ* = 0.938), elaboration connectives (ELAB; *λ* = 0.592), and split infinitives (SPLIT; *λ* = 0.477).

This dimension captures a narrower, more lexically specific cluster of elaborative and predicative complexity markers. It shows a significant but modest effect (*χ*^2^ = 7.58, *p* = 0.023, Kendall’s *W* = 0.158); human translation scores significantly lower than NMT, while neither of the other two pairwise contrasts survives Holm correction.

#### 4.2.7 Summary across dimensions

Across all five dimensions (Figure 3), the clearest and most robust pattern is HT’s distinctiveness from both machine systems on discourse cohesion (D3). The remaining four dimensions are individually weaker; several are significant at the conventional α = 0.05 level, and—notably—Dimension 1 reverses the naive expectation that machine output is uniformly “simpler”: here, both machine systems, and V4Pro in particular, display greater nominal/lexical density than HT, which plausibly reflects source-language shining-through rather than genuine simplification.

**Figure 3:**
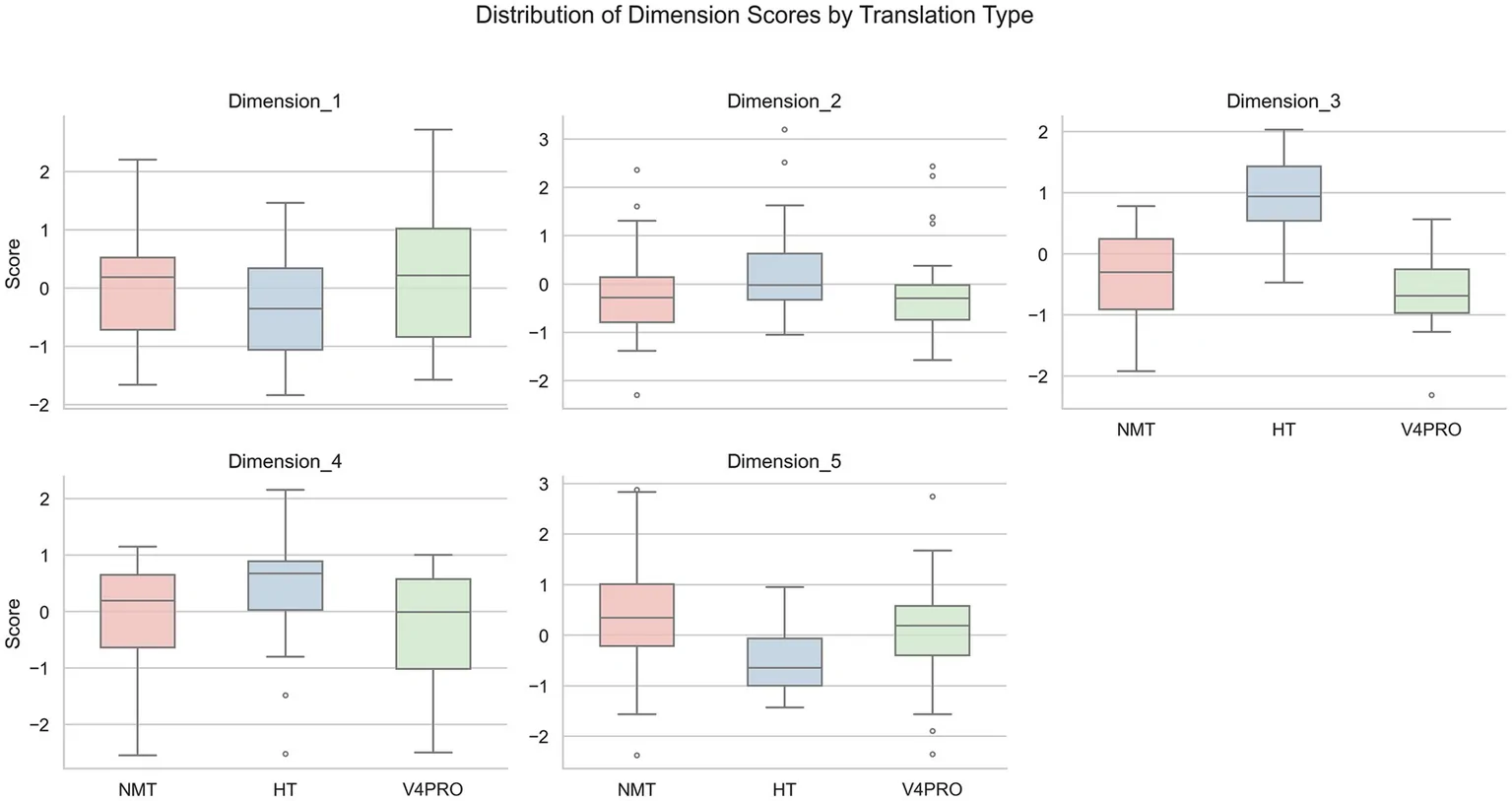
Distribution of dimension scores across translation types.

#### 4.2.8 Non-circularity check for the factor structure

The 24-feature exploratory set (Section 3.3) was selected precisely because these features differ across the three translation conditions, and the resulting factor scores are then tested again for differences among those same conditions; accordingly, the significance tests reported in Table 4 carry an element of circularity. To evaluate how much this matters, we re-derived the factor structure from an independently selected, variance-based (non-group-contrast) feature subset of the same size (24 features) and then compared the two solutions.

Under the group-contrast-based selection, all five dimensions were significant (5/5). Under the independent, variance-based selection, only 3 of 5 raw factors were significant. However, after Procrustes rotation matching each variance-based factor to its best-corresponding group-contrast dimension, all five matched dimensions were again significant (5/5). One-to-one congruence coefficients between matched dimension pairs were 0.697 (D1), 0.707 (D2), 0.382 (D3), 0.703 (D4), and 0.535 (D5). The direction of the pairwise group contrasts was replicated on 12 of 15 matched comparisons.

We read this pattern as follows. D1, D2, and D4 show reasonably strong one-to-one congruence (0.70–0.71), and an independent selection procedure corroborates them. D5’s congruence (0.535) is more modest. D3—the dimension with by far the largest effect size in the main solution—shows the lowest congruence (0.382) despite remaining significant after Procrustes matching; we interpret this as indicating that the discourse-cohesion contrast itself is robust (it reappears under an independent selection and the group differences replicate in direction for most comparisons), while the specific factor loading pattern that defines it is more sensitive to which features enter the analysis than the other dimensions are. In light of this mixed pattern, we present the five-factor MDA solution as an exploratory characterization of the corpus’s functional structure rather than as a fully confirmed dimensional model. We recommend that the discourse-cohesion contrast (Section 4.2.4)—rather than the full loading structure of Dimension 3—be treated as the most secure finding to carry forward into interpretation.

#### 4.2.9 Pairwise contrasts

Because an omnibus Friedman test indicates only that the three conditions differ somewhere, and does not by itself justify any specific pairwise interpretation, Table 6 reports Holm-corrected Wilcoxon signed-rank tests, effect sizes, and bootstrap confidence intervals. These are provided for every HT–NMT, HT–V4Pro, and NMT–V4Pro contrast on each dimension.

**Table 6:** Post-hoc pairwise comparisons.

Dimension	Pair	Wilcoxon *p*	Effect *r*	Median diff.	95% CI	Holm *p*	Sig.
D1	HT–NMT	0.001	−0.747	−0.275	[−0.743, −0.109]	0.009	**
D1	HT–V4Pro	<0.001	−0.807	−0.647	[−1.073, −0.431]	0.003	**
D1	NMT–V4Pro	0.019	−0.540	−0.357	[−0.515, 0.126]	0.107	n.s.
D2	HT–NMT	0.006	0.627	0.313	[0.232, 0.677]	0.041	*
D2	HT–V4Pro	0.018	0.547	0.255	[−0.009, 0.533]	0.107	n.s.
D2	NMT–V4Pro	0.900	−0.033	0.054	[−0.268, 0.191]	0.900	n.s.
D3	HT–NMT	<0.001	0.993	1.264	[0.868, 1.450]	<0.001	***
D3	HT–V4Pro	<0.001	1.000	1.346	[1.204, 1.786]	<0.001	***
D3	NMT–V4Pro	0.001	0.747	0.327	[0.182, 0.431]	0.009	**
D4	HT–NMT	0.002	0.700	0.578	[0.123, 0.744]	0.016	*
D4	HT–V4Pro	0.004	0.647	0.516	[−0.028, 1.025]	0.035	*
D4	NMT–V4Pro	0.375	0.213	0.075	[−0.158, 0.474]	0.750	n.s.
D5	HT–NMT	0.001	−0.727	−0.863	[−1.399, −0.177]	0.011	*
D5	HT–V4Pro	0.056	−0.447	−0.381	[−1.071, 0.031]	0.170	n.s.
D5	NMT–V4Pro	0.042	0.473	0.422	[0.083, 0.759]	0.170	n.s.

*p < 0.05, **p < 0.01, ***p < 0.001 (Holm-corrected); n.s. = not significant.

Three patterns emerge. First, on D1, D2, and D4, HT differs significantly from both machine systems (with the HT–V4Pro contrast on D2 the sole exception, which does not survive Holm correction), while NMT and V4Pro are statistically indistinguishable from each other. Second, D3 (discourse cohesion) represents the only dimension on which all three pairwise contrasts are significant after correction, showing a strict ordering of HT > NMT > V4Pro. Third, for D5, only the HT–NMT contrast survives correction; the apparent NMT–V4Pro difference (raw *p* = 0.042) does not survive Holm correction and should not be treated as established.

### 4.3 Distance calculation and t-SNE visualization (RQ3)

The raw distances in Table 7 and Figure 4 show that, in the five-dimensional stylometric space, DeepSeek V4Pro and Google NMT are more stylistically similar to each other (mean distance = 2.988) than either is to human translation (HT–NMT = 3.251; HT–V4Pro = 3.408).

**Table 7:** Mean pairwise Euclidean distances in five-dimensional factor score space.

Pairwise comparison	Mean distance	Std. Dev.
HT–NMT	3.251	0.897
HT–V4Pro	3.408	0.875
NMT–V4Pro	2.988	1.026

**Figure 4:**
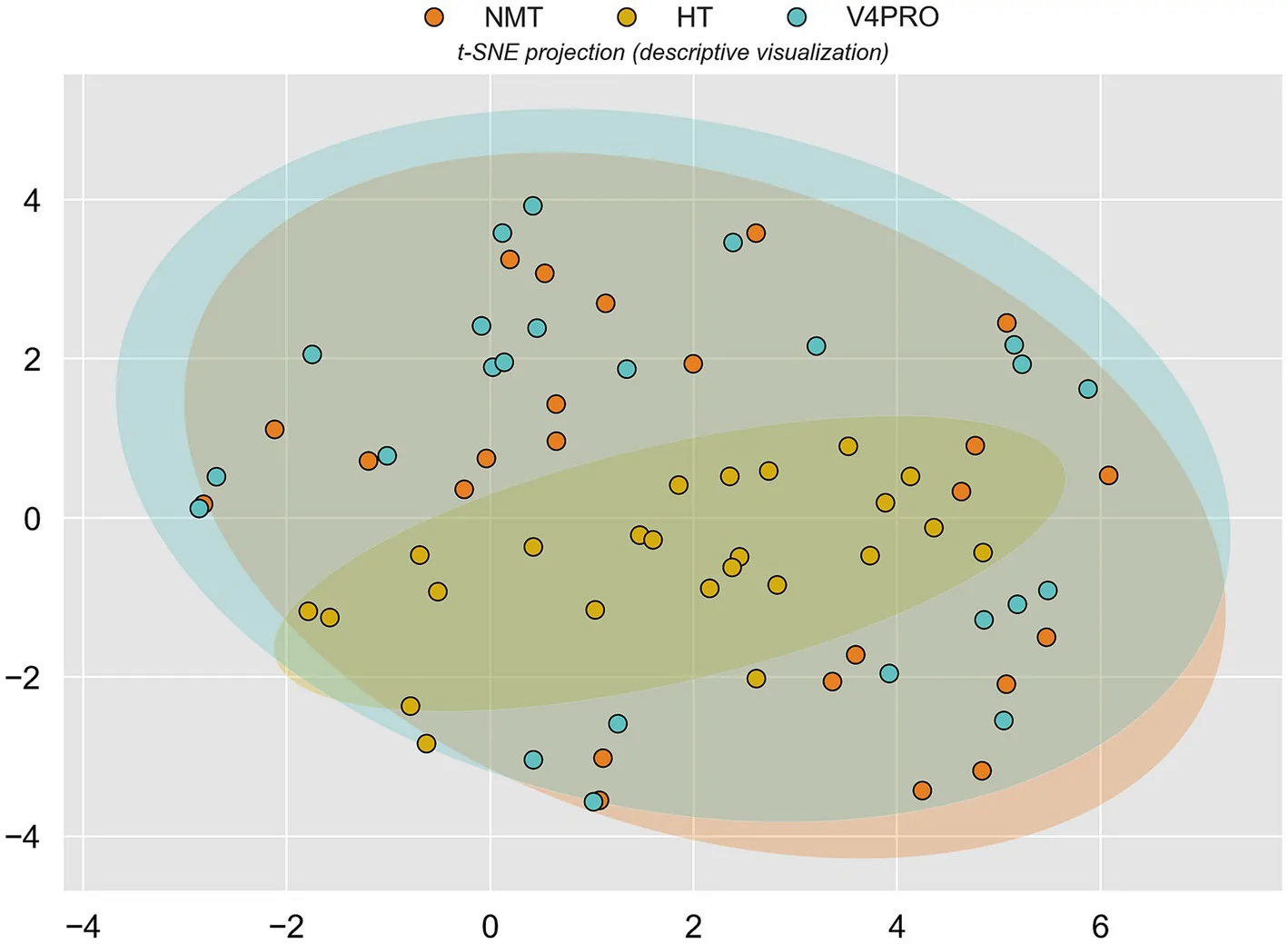
t-SNE projection of factor scores with confidence ellipses.

Resampling whole chunks (rather than individual windows) preserves the paired design and provides a source-controlled estimate of sampling uncertainty. Both differences that compare a human–machine distance against the machine–machine distance exclude zero (HT–NMT vs. NMT–V4Pro: 95% CI [0.097, 0.502]; HT–V4Pro vs. NMT–V4Pro: 95% CI [0.295, 0.653]), so the finding that NMT and V4Pro sit reliably closer to each other than either does to HT is statistically supported, not merely a pattern in the point estimates (Table 8). By contrast, the difference between the two human–machine distances themselves spans zero (95% CI [−0.367, 0.028]), so we do not claim that V4Pro is reliably closer to, or farther from, HT than NMT is; HT is approximately equidistant from the two machine systems, which are themselves closer to one another than either is to HT. This finding provides a direct answer to RQ3: the data support the existence of a shared machine-translationese region distinct from institutional human translation, but they do not support a stronger claim that V4Pro is detectably more (or less) human-like than NMT within this five-dimensional space.

**Table 8:** Chunk-level bootstrap analysis of pairwise distances and their differences.

Quantity	Observed	Bootstrap mean	2.5%	97.5%
HT–NMT	3.251	3.215	2.895	3.526
HT–V4Pro	3.408	3.378	3.085	3.655
NMT–V4Pro	2.988	2.915	2.612	3.195
(HT–NMT) − (HT–V4Pro)	−0.156	−0.163	−0.367	0.028
(HT–NMT) − (NMT–V4Pro)	0.263	0.300	0.097	0.502
(HT–V4Pro) − (NMT–V4Pro)	0.420	0.463	0.295	0.653

## 5 Discussion

### 5.1 The distinction between humans and machines: a persistent but more narrowly supported boundary

The findings of this study reveal a pattern that, although Google NMT and DeepSeek V4Pro differ considerably in design, both systems systematically diverge from professional human translation on discourse cohesion and causal-deictic linking (D3) and, more modestly, on nominal/lexical density (D1), nominalization and wh-subordination (D2), and lexical diversity/dynamic aspect (D4).

Building on Sheng and Kong's (2023) observation that NMT fails to replicate the discourse agency of institutional voice, this finding suggests the shortcoming is not limited to modal choices or stance markers but extends to the structural scaffolding of argumentation itself. Since government white papers build sustained arguments—they state a position, give reasons, address objections, and connect claims—institutional translators reproduce this logical scaffolding through explicit linking devices. Both machine systems, trained to prioritize sentence-level naturalness over document-level argumentative structure, under-represent these connective elements even though, as documented in Section 3.1.2, both had document-level source context available during generation. Read through a cognitive-processing lens (Sweller, 1988; Baddeley, 1990), we suggest—as a hypothesis for future testing rather than a demonstrated outcome of the present design, which included no reader experiment—that explicit cohesive markers may reduce the working-memory burden a reader carries while integrating successive propositions into a coherent argument. Furthermore, their removal could push readers of government white papers toward inferring logical relationships the source text intended to state explicitly.

Dimension 1 (nominal and lexical elaboration) complicates a simple “machines simplify” narrative: human translation scores significantly lower than both machine systems here, particularly V4Pro. Reading this result alongside Li and Hu's (2024) discussion of “shining through,” a plausible account emerges: the Chinese source text is itself nominally dense, and machine systems—which optimize translation closer to the source at the phrase and sentence level—carry more of that nominal density through into English; by contrast, professional human translators characteristically re-verbalize dense nominal strings into a more idiomatic English clause structure.

### 5.2 The specificity of LLM-generated translation

DeepSeek V4Pro occupies substantially the same broad “machine-translationese” region as Google NMT, not a clearly separate one. The RLHF-driven-hedging account is, in our view, a theoretically motivated and worthwhile hypothesis for a study designed specifically to test it—for instance by comparing multiple RLHF-tuned LLMs against multiple NMT systems.

These interrelated factors offer a plausible explanation for the machine–human divergence, chiefly evident in D3 and to a lesser extent in D1, D2, and D4. First, the matter of pre-training data composition is addressed. NMT systems are typically trained on curated, largely formal parallel corpora (UN documents, parliamentary records, news). In contrast, LLMs are pre-trained on web-scraped and conversational data that emphasizes informal, dialogue-oriented language, patterns that later fine-tuning does not fully suppress. Both systems’ training objectives reward sentence-level fluency and naturalness over document-level argumentative scaffolding. This would predictably erode explicit cohesive marking regardless of whether the underlying architecture is encoder–decoder or decoder-only. Second, divergent handling of source-language nominal density: NMT’s closer optimization to source-level correspondence and LLMs’ more variable, sampling-influenced generation may both allow more source-nominal-structure transfer than a human translator’s active re-verbalization.

Situating these findings within the cognitive-load and AI-literacy literature still strengthens their practical relevance, provided the connection is stated as a hypothesis. Following Yao and Fan's (2026a) Critical AI Literacy framework, the discourse-cohesion gap documented here implies that translators and post-editors working with machine output need the evaluative capacity to detect and restore missing connective structure, not simply to check for outright mistranslation. Following Yao and Fan (2025), the cognitive effort of restructuring under-cohesive machine output into institutionally calibrated, well-linked prose is a plausible candidate for a measurable, distinct form of cognitive load, and future work could apply their scale directly to post-editors working with the DeepSeek/NMT outputs analyzed here. Finally, following Yao and Fan (2026b), how a translator responds to this register variation—critically intervening to restore cohesion, versus passively accepting the model’s default—plausibly depends on the translator’s own self-efficacy in evaluating AI-generated text, a psychological variable that the present structural findings offer a natural basis for extending.

### 5.3 Significance for translation practices in institutional contexts

The results offer concrete practical implications for AI translation in Chinese government communications at three levels.

First, post-editing should be tailored to the one robustly demonstrated weakness common to both systems—under-elaborated discourse cohesion—rather than applied uniformly across every stylistic dimension: both NMT and DeepSeek V4Pro output would benefit from targeted discourse-cohesion repair (reinserting causal, contrastive, and resultative connectors), while claims about system-specific weaknesses beyond this (e.g., a distinctive V4Pro hedging problem) should await further, purpose-built evidence.

Second, matching engines to sub-genres of government communication remains a reasonable, if now more tentative, implication of the D3 finding: since HT’s advantage is clearest and most robust on discourse cohesion specifically, texts whose communicative success depends heavily on explicit argumentative scaffolding (position papers, policy justifications) may benefit most from human translation or focused post-editing, regardless of which machine system produced the draft.

The value of human translation is best stated as a difference in stylistic profile rather than a general claim of translation-quality superiority. This study does not evaluate translation accuracy, fidelity, or acceptability, and the human translations examined here are published, post-edited texts, whereas the machine translations are unedited raw outputs. What the data support is that professional human translation more closely reproduces the established stylistic profile of institutional discourse—most clearly its density of explicit cohesive marking—than either machine system’s raw output does. In the contexts where communicative stakes are highest—treaty negotiations, formal policy announcements, legally binding declarations—this closer match to institutional convention is itself meaningful, and we do not need to claim, nor can we claim from this design, that HT is a higher-quality translation in the fidelity sense.

## 6 Conclusion

This study compared stylistic profiles of Chinese government white paper translations by DeepSeek V4Pro, Google NMT, and human translators using a rolling stylometry corpus (72 aligned windows) and multi-feature analysis. Classification was above chance but modest and model-dependent; Naive Bayes and Random Forest were most reliable (accuracy ~0.6–0.87 vs. 33.3% baseline), supporting a detectable stylistic signature. Five significant stylistic dimensions emerged; discourse cohesion/causal-deictic linking (D3) was strongest, while other dimensions were weaker. Human translations differed from both MT systems; the two MT systems differed only on discourse cohesion. Distance analysis placed DeepSeek and Google NMT closer to each other than either was to human translation, with no reliable difference between the two human–machine distances.

The findings provide support for a machine-translationese shared by NMT and LLM output, which is most clearly and robustly expressed as reduced discourse cohesion relative to institutional human translation. We situate this structural account within a cognitive-processing framework connecting reduced cohesive marking in machine-mediated institutional text to reader processing load and stance attribution, while treating that link, and any RLHF-based mechanistic account of LLM-specific patterns, as a hypothesis for future work.

### 6.1 Limitations

Several limitations of this study must be acknowledged. First, because the analysis compares post-edited human translations with unedited machine-generated texts, some identified differences may stem from editorial changes rather than raw human-translation style; future studies using controlled comparisons of unedited human drafts would sharpen this boundary. Second, although our diachronic stability checks found the pre-2013 HT baseline substantially stable in the syntactic/modal features driving our factor structure, it is not a full substitute for a time-matched HT corpus. Residual diachronic confounding cannot be fully excluded. Third, the corpus is limited to one text type—government white papers—and one producer—the State Council Information Office—so this restricts generalizability to other government document types, sources, or language pairs. Fourth, it must be considered that DeepSeek V4Pro represents only one iteration within a rapidly evolving model family; future versions will likely exhibit different stylistic characteristics as training data, architecture, and RLHF procedures continue to change. Fifth, the numbered-sentence prompting protocol used for DeepSeek V4Pro, while it enforces a checkable source correspondence, is a specific prompting choice; different prompting strategies (e.g., paragraph-level or few-shot prompting) could plausibly shift the discourse-cohesion and nominal-density patterns identified here, and this should be tested directly in future work.

### 6.2 Future research directions

First, original research using raw, unedited human translations produced in controlled settings would sharpen the boundary between human and machine translation style. Second, cross-system comparison (GPT-6, Claude, Gemini, and newer models) would help chart which stylistic features are LLM-general versus system-specific, and would allow the discourse-cohesion pattern identified here to be tested for generality across RLHF-tuned systems, alongside a purpose-built test of the hedging/RLHF hypothesis that the present design could not establish. Third, combining this stylistic analysis with qualitative, example-level examination, with direct measurement of post-editors’ cognitive load, critical AI literacy, and self-efficacy when working with these systems’ output would connect the structural findings reported here to their actual processing and workplace consequences. In addition, the compact, interpretable 21-feature classification set reported here (Appendix B) could serve as a transparent baseline for future work on detecting machine-translated or LLM-translated institutional text; a systematic evaluation of its performance against embedding-based detection systems is a natural extension of the present study. Finally, complementing the structural and functional features used here with affective and semantic dimensions (e.g., emotional tone) would provide a fuller stylistic characterization of institutional translation and is a promising direction for future work.

## Data Availability

The original contributions presented in the study are included in the article/Supplementary material, further inquiries can be directed to the corresponding author/s.
