# Supplementary material for: Differentiating DeepSeek AI-generated translations from NMT translations and human translations within Chinese white paper discourse: a computer-based method for analyzing writing style
Source: Front Psychol. 2026 Oct 26;17:1931933. doi: 10.3389/fpsyg.2026.1931933 (PMC13745704; doi:10.3389/fpsyg.2026.1931933)
Supplement: Supplementary file 1 [file Supplementary_file_1.docx]

Appendix A: Corpus Composition Details

| Sub-corpus | Label | Time Period | Types | Tokens | Windows |
| --- | --- | --- | --- | --- | --- |
| Human Translation | HT | Pre-2013 | 7,140 | 109,335 | 24 |
| Google NMT | NMT | 2026 | 6,055 | 109,130 | 24 |
| DeepSeek V4Pro | V4Pro | 2026 | 6,326 | 108,274 | 24 |
| Diachronic check: Human Translation (post-2014) | HT2014 | 2014–2026 | 11,273 | 255,028 | 56 |

Analysis rolling stylometry parameters: window size = 5,000 words; step = 4,500 words; overlap = 500 words; windows source-aligned across HT, NMT, and V4Pro via HT-based chunk boundaries (Section 3.1.3).

Appendix B: Final Feature Sets

The exploratory (MDA) feature set consists of 24 features, selected via paired Friedman test across the full 72-window sample (30 of 139 features significant after Bonferroni correction) followed by correlation-based pruning (|r| > 0.8; 6 features removed).

The feature list is LDE, AWL, RB, TTR, SPLIT, INother, VIMP, ACT, RP, NN, QUAN, ELAB, PP1P, PP3all, WHSC, NCOMP, NOMZ, JJPRother, PROG, JJAT, CUZ, CC, PIT, DEMO.

The classification feature set consisted of 21 features, selected via the same procedure applied only within the 57-window training partition (27 of 139 features significant after Bonferroni correction), followed by the same |r| > 0.8 correlation-based pruning:

The feature list is LDE, AWL, RB, TTR, SPLIT, VIMP, INother, ACT, RP, PP1P, NN, QUAN, ELAB, WHSC, NCOMP, PP3all, JJPRother, NOMZ, CUZ, JJAT, CC.

Features removed for redundancy in both pipelines (|r| > 0.8): IN, JJATother, PP1all, PP3t, RBother, WHSCother.

Feature key (features not already defined in the main text): TTR = type-token ratio; INother = other/residual prepositions; PP1P = first-person plural pronouns; JJPRother = residual (other) predicative-adjective category; VIMP = residual verbal/stance category.

Appendix C: Factor Analysis Technical Details

Adequacy tests: KMO = 0.602 (acceptable; Kaiser 1974); Bartlett's p < .001 (confirms matrix factorability).

Extraction method: Principal axis factoring.

Rotation: Varimax (orthogonal), maximising simple structure.

Factor score computation: Regression method (Thomson scores), z-standardised input features.

Elbow detection: The Kneedle algorithm was applied to the normalised eigenvalue sequence; a formal elbow was identified at Factor 5; five factors were retained.

Dimension-level significance testing: paired Friedman test across the three translation types, matched by ChunkID; Kendall's W reported as effect size; Holm-corrected Wilcoxon signed-rank tests reported for all pairwise contrasts.

Loading threshold: |λ| ≥ 0.30 for dimension interpretation.

For non-circularity validation, the five-factor solution was re-derived from an independently, variance-based (non-group-contrast) selected 24-feature subset and matched to the main solution via Procrustes rotation; one-to-one congruence and direction-of-effect agreement are reported in Section 4.2.10.

Appendix D: Computational Pipeline Summary

The pipeline is structured in six stages: (1) data loading, label assignment, and construction of source-aligned, minimally overlapping (500-word overlap) rolling-window chunks keyed to a common ChunkID across HT, NMT, and V4Pro; (2) two independent feature-selection procedures — an exploratory pipeline using the full 72-window sample (paired Friedman test with Bonferroni correction, then correlation-based filtering at |r| > 0.8) for the multidimensional analysis, and a classification pipeline restricted to the 57-window training partition only, using the same two-step procedure; (3) machine-learning classification (5 classifiers; group-aware 80/20 train/test split by ChunkID; 5-fold GroupKFold cross-validation within the training partition; nested cross-validation with per-fold feature re-selection; 50 repeated group-aware splits with and without a one-chunk training buffer); (4) multidimensional analysis (StandardScaler → FactorAnalyzer with varimax rotation → paired Friedman test with Kendall's W on dimension scores → Holm-corrected Wilcoxon post-hoc tests → Procrustes-based non-circularity check against an independently, variance-based selected feature subset); (5) distance computation (scipy.spatial.distance.cdist, metric = 'euclidean'), t-SNE visualisation (sklearn.manifold.TSNE, n_components = 2, perplexity = 30), and a chunk-level bootstrap (5,000 resamples) of pairwise distances and their differences; (6) diachronic stability check (Mann–Whitney U with Bonferroni correction, pre-2013 vs. post-2014 HT).

Appendix E: A Representative API Call

python

import os

from openai import OpenAI

api_key = os.environ.get("DEEPSEEK_API_KEY")

client = OpenAI(api_key=api_key, base_url="https://api.deepseek.com")

numbered_source = "1. 20 世纪50 年代，中非经贸合作以贸易和对非援助为主。 \n2. 在双方共同努力下，合作领域不断拓宽，合作内容日益丰富。"

user_content = f"""The following is a list of sentences split from a paragraph

of Chinese text, with each sentence on a separate line and numbered. Please

translate each sentence into English. Strictly output the translations

sentence by sentence with the same numbering as the original, in the format

"Number. Translation". Do not output the original text, do not merge multiple

sentences into one line, do not split one sentence into multiple lines or

multiple numbers, do not omit or add numbers, and do not output anything

outside the numbered list (no explanations, prefixes, or suffixes).

{numbered_source}"""

response = client.chat.completions.create(

model="deepseek-v4-pro",

messages=[

{"role": "system", "content": "You are a helpful assistant"},

{"role": "user", "content": user_content},

],

stream=False,

reasoning_effort="high",

extra_body={"thinking": {"type": "enabled"}},

temperature=0

)

print(response.choices[0].message.content)
